# Implementation processes of social network interventions for physical activity and sedentary behavior among children and adolescents: a scoping review

**DOI:** 10.1186/s12889-024-18615-6

**Published:** 2024-04-22

**Authors:** Jose Petro-Petro, Carlos Mario Arango-Paternina, Fredy Alonso Patiño-Villada, Jhon Fredy Ramirez-Villada, Ross C. Brownson

**Affiliations:** 1 Instituto de Educación Física, Universidad de Antioquia, Carrera 75 Nº 65-87 - Bloque 45, Medellín, Colombia; 2 Departamento de Cultura Física, Universidad de Córdoba, Montería, Colombia; 3 Research Group on Physical Activity for Health (AFIS), Instituto Universitario de Educación Física y Deportes; Universidad de Antioquia, Ciudadela Robledo, Medellín, Colombia; 4 Prevention Research Center, Brown School, Washington University, St. Louis, MO USA; 5 Department of Surgery (Division of Public Health Sciences) and Alvin J. Siteman Cancer Center, Washington University School of Medicine; Washington University, St. Louis, MO USA

**Keywords:** Health-related behaviors, Social network analysis, Dissemination and implementation research, Social norm, Social support, Social influence, Social pressure, Social modeling

## Abstract

**Background:**

The characteristics of the implementation process of interventions are essential for bridging the gap between research and practice. This scoping review aims to identify the implementation process of social network interventions (SNI) to address physical activity and sedentary behaviors in children and adolescents.

**Methods:**

The scoping review was conducted adhering to the established guidelines. The search was carried out in the ERIC, EBSCO, EMBASE, SCOPUS, and Lilacs databases in April 2023. Social network intervention studies in children and adolescents were included, addressing physical activity or sedentary behaviors. Replicability (TIDieR), applicability (PRECIS-2), and generalizability (RE-AIM) were the explored components of the implementation process. Each component was quantitatively and separately analyzed. Then, a qualitative integration was carried out using a narrative method.

**Results:**

Most SNI were theoretically framed on the self-determination theory, used social influence as a social mechanism, and used the individual typology of network intervention. Overall, SNI had strong replicability, tended to be pragmatic, and three RE-AIM domains (reach, adoption (staff), and implementation) showed an acceptable level of the generalizability of findings.

**Conclusions:**

The analyzed SNI for physical activity and sedentary behaviors in adolescents tended to be reported with high replicability and were conducted pragmatically, i.e., with very similar conditions to real settings. The RE-AIM domains of reach, adoption (staff), and implementation support the generalizability of SNI. Some domains of the principles of implementation strategies of SNI had acceptable external validity (actor, action targets, temporality, dose, and theoretical justification).

**Supplementary Information:**

The online version contains supplementary material available at 10.1186/s12889-024-18615-6.

## Background

Dissemination and implementation (D&I) research is a comprehensive approach focused on translating scientific knowledge into practice and policy [1]. D&I science is a field with constant development in terms of theories, frameworks, and methods helpful in bridging the gap between what is known from research and what is implemented in practice. In this process, scientific evidence of the effectiveness of interventions is as crucial as implementing these interventions. Therefore, D&I investigates for whom the scientific evidence could be helpful, in which contexts it would work, and what mechanisms would explain its usefulness [2].

In this regard, and from the D&I research perspective, the exploration of the implementation process places a significant focus on external validity [3]. External validity may be examined by considering different dimensions such as replicability, applicability, and generalizability. Here, replicability analysis focuses on those methodological features reported in sufficient detail so that one can ascertain whether the study may be replicated in a manner similar to the original version [4]. Applicability is understood as an attribute that informs whether the intervention study is closer to “real world” conditions (pragmatic) or idealized conditions (explanatory) [5]. Generalizability refers to the extent to which a study’s findings may translate into practice in settings situations and populations [6].

In the field of social determinants of health-related behavior, interpersonal relationships are the foundation of social network intervention since social network dynamics unfold from social relationships [7]. The associations between social networks and health behaviors are well-established [8–10], and particularly for physical activity (PA) and sedentary behavior (SB), the content of social relationships such as social norms, social support, and social influence are the mechanisms that help to explain how social networks affect these behaviors [11–15].

Social network interventions (SNI) have shown a growing interest, and one of its tenets is using network data to accelerate behavior change or improve organizational performance within the network [16]. Four types of SNI have been proposed in the literature: an individual approach aimed to identify leaders to promote behavior change, and three network-based approaches: induction, segmentation, and alteration [16]. The evidence synthesis for social network studies in adolescents indicates that the effectiveness of SNI in changing health-related behaviors has shown promising results in different health outcomes and populations and suggests that the strongest evidence of effectiveness has been for the individual approach [8]. However, implementation elements need to be clarified or provided in detail in this body of literature, including social mechanisms and theoretical foundations [8], since these elements are critical components for D&I science [17] and to understand how social dynamics affect behavior [16]. In addition, the optimal way to apply different SNI approaches in interventions that address health-related behaviors is still unknown [8]. It is necessary to study further SNI characteristics [13, 14], specially describing the complexity and contextual factors that are essential from the D&I perspective, such as personal, cultural, social, environmental factors, among others [18].

Considering the deficient evidence about and the complexity and breadth of the implementation process of SNI, this scoping review aimed to evaluate and synthesize the implementation process of SNI to modify PA and SB in childhood and adolescence. For this purpose and following the suggestion made for evidence synthesis studies for external validity [19], the integration of replicability, applicability, and generalizability, was made recognizing that social intervention are complex and multilevel, and should offer enough detail to apply these implementation strategies in different contexts [20]. For this reason, the typology purposed by Proctor et al. [20] was used to integrate key components of implementation strategies with external validity dimensions. This integration would provide greater clarity of how scientific advances about social network interventions could inform future translation into practice. This integration, and therefore, the characterization of the implementation strategies.

## Methods

This scoping review was conducted following the methodological protocols established by the Jhoana Briggs Institute (JBI) [21], whose guides are based on the population, concept, and context framework (PCC) [21]. This review was focused on the population of children and adolescents; the concept used was primary studies of social network interventions in PA and SB. No restrictions were made on the context. The report was elaborated following the PRISMA Extension for Scoping Reviews (PRISMA-ScR) Checklist and Explanation [22]. The implementation process was analyzed exploring different aspects of SNI, including theoretical support, interpersonal relationships fostered in these interventions, types of social network interventions implemented, and how replicable, applicable, and generalizable were the SNI. The scoping review protocol was registered in Open Science Framework as part of the transparency of the study (Registration DOI: 10.17605/OSF.IO/XS3RU).

### Eligibility criteria

This scoping review considered social network intervention articles regardless of network intervention strategy. Studies should consider multiple relationships among individuals in the social network and should have PA and SB in children and adolescents in any context as outcome variables. Social network interventions were defined as those studies that intentionally use social network data to generate behavioral changes among individuals within the network. The studies must have measured social relationships and used these measurements to intervene, regardless of the network approach used. Those studies based only on simulated interventions such as agent-based modeling were excluded. Also, following the operational definition of SNI, studies about only on dyadic, or triad relationships were excluded since the design of these types of interventions is not based on network data.

### Types of sources

Any primary intervention study was considered regardless of the design or approach used (quantitative, qualitative, or mixed studies).

### Search strategy

The search strategy was aimed at locating published studies. The search strategy was not limited by study design, year, region, publication time, or language. Two reviewers were going through the process of identifying the most appropriate terms through an initial search based on previous evidence syntheses. The search strategy was tailored for each database, including all specified keywords and index terms related to PA, SB, and SNI. The reference list of all included sources of evidence was screened for additional studies.

For the complete search, the following electronic databases were used: ERIC, EBSCO, EMBASE, SCOPUS, and Lilacs. The search for information was done in April 2023. For each variable, related terms were used and included physical activity, sport, sedentary behavior, sedentary behaviour, sitting, screen time, screen use, sedentary time, sedentary lifestyle, social network analysis, network intervention, network-based intervention, social network intervention, and friends intervention. The search equation in each database was specified in Supplementary Table S1.

### Study/source of evidence election

Once the search was done, the Rayyan bibliographic manager [23] was used to download, organize the bibliography, and eliminate duplicate references. Two reviewers (JP and CA) independently reviewed titles and abstracts for evaluating and selecting evidence, considering the inclusion criteria to select potential review sources. Then, they examined the full text of the studies. Any disagreement between the two reviewers was settled by consensus.

### Data extraction

Two reviewers (JP and CA) independently extracted the data from the articles in a template developed following the JBI guidelines [24], and previously pilot-tested. According to the review question, the template was designed with precise information about participants, concept, context, methodology, and essential results. Any disagreement (about 10% for the entire extraction process) was resolved by consensus. The minimum data for the data extraction form included complete citation data (author, year, title, journal), country, participants/population, sample characteristics, outcome variables, approach for social network intervention, context, study-oriented basic theory, the content of fostered interpersonal relationships, details of the intervention process, results (primary and secondary), and conclusions.

The analyzed external validity dimensions included replicability, applicability, and generalizability (Supplementary table S2). Replicability was analyzed with the Template for Intervention Description and Replication tool, TIDieR [25], covering 12 items (Supplementary Table S2). TIDieR score in each item was assessed utilizing 1 = reported and 0 = no reported. The total score of each study was obtained by adding the value of each item, and the score of each item was obtained by adding the value in each study. The Pragmatic-Explanatory Continuum Indicator Summary model, PRECIS-2 [5], was used to evaluate the applicability of the interventions, considering nine items (Supplementary Table S2). Each domain was scored using a 5-point Likert scale: (1) Very explanatory, (2) Rather explanatory, (3) Equally pragmatic and explanatory, (4) Rather pragmatic, (5) Very pragmatic [5]. Generalizability was explored with the reach, effectiveness, adoption, implementation, and maintenance framework (RE-AIM) (Supplementary Table S2) [26]. RE-AIM score in each item was measured as follows: “Not reports = 0”, “Accurate reported = 1”, and “Misreporting = 2”. The percentage of each dimension is reported by applying the following formula (criteria sum * 100/Number of items in each dimension).

### Data analysis and presentation

A qualitative analysis of the evidence of the implementation process was carried out using a narrative method. The focus of the present scoping review was the characteristics of the implementation process of the SNI. We did not quantify or measure the effectiveness of the interventions. The process evaluation was analyzed using the TIDieR, PRECIS-2, and RE-AIM tools. The data are represented in tables and graphs with their respective qualitative analyses. As an intent to integrate findings from these three tools, the principles of implementation strategies proposed by Proctor et al. [20]. were used to indicate how replicable, applicable, and generalizable the SNI were in terms of the seven dimensions: actor, the action, action targets, temporality, dose, implementation outcomes, and theoretical justification.

## Results

**Fig. 1:**
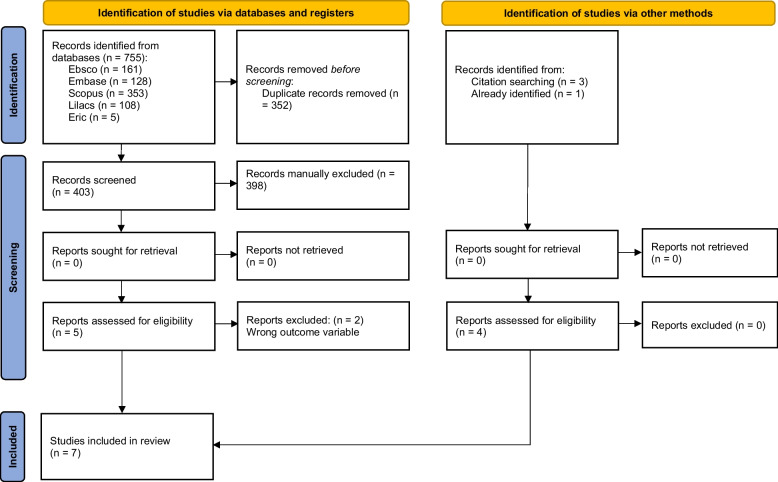
PRISMA flow diagram illustrating the searching and selection process

After the search, 755 citations were identified from all databases. After removing duplicates, we screened 403 citations in the title and abstract; five studies met the inclusion criteria for full paper revision [27–31]. After this, two studies were excluded because PA or SB was not measured as the outcome variable in one study [31], and the other was not a SNI [30]. We identified and included three citations found by manual search [32–34], and one study was included for the researchers’ knowledge [35]. In total, seven studies were analyzed for this scoping review. Figure 1 shows a flow diagram of publication identification, screening, eligibility assessment, and inclusion.

### Characteristics of the studies

The seven studies were published between 2012 and 2022, and six were published in the last six years (from 2017 to 2022), indicating the relative newness of the social network approach to address PA and SB in children and adolescents. All studies were developed in the school context and were conducted in high-income countries: three in England [32–34], two in the Netherlands [27, 28], one in the United States [35], and one in Italy [29]. Three studies measured both PA and SB by accelerometry [32–34]; four studies measured only PA, three by accelerometry [27–29], and the other one by questionnaire [35]. Six studies were conducted using the randomized control trial design; the other used a quasi-experiment design [35]. The age range of the population was 9–16 years old, with a total of 4046 participants in all the studies. Characteristics of the included studies are shown in Table 1.

**Table 1:** Characteristics of the included studies

Study, country	Methods	Details of the intervention
Proestakis, 2018 [29] Italy	**Study design**: Cluster RCT. **Sample characteristics**: 349 children (9–11 years old), 177 females and 172 males, from 19 fifth-grade elementary school classes in the same region. IG “Individual regards” (*n* = 87); IG “Direct reciprocity” (*n* = 50; IG “Indirect reciprocity” (*n* = 61); IG “Team rewards” (*n* = 75); CG (*n* = 76). **PA and SB measurements**: Accelerometry. SB: No measured.	**Supporting theory**: Social network theory. **Social function promoted**: Social norms, social influence, and social pressure. **Intervention**: 7-week intervention to different social incentives in two experimental phases. Five conditions groups were established—four groups in intervention. Students were matched with classmates of the same sex and strong ties (best friends). Three intervention conditions of social-rewards schemes were established: Direct reciprocity, indirect reciprocity, and team rewards, in which classmates’ rewards depend on the PA of two friends either reciprocally (directly or indirectly) or collectively. Additionally, one intervention condition of individual rewards was established. The control group was established with a random reward. **SNI approach**: Induction and alteration. **Effects**: Compared with a random-rewards control, social-rewards schemes have an overall significantly positive effect on PA.
Bell, 2017 [32] England	**Study design**: Exploratory RCT. **Sample characteristics**: 928 adolescents (12–13 years old) from six co-educational comprehensive schools. IG (*n* = 462); CG: *n* = 466). **PA and SB measurements**: Accelerometry.	**Supporting theory**: DOI. **Social function promoted**: Social influence and social support. **Intervention**: 10-week intervention, informed by a focus group with students, interviews with professional persons, and document review. Peer supporters were identified by nominations and then trained to play a supporter role within their classrooms to encourage peers in PA and decrease sedentary time. The control group received no intervention. **SNI approach**: Individual. **Effects**: No effects on PA or SB
Woudenberg, 2018 [28] Netherlands	**Study design**: Cluster RCT. **Sample characteristics**: 190 adolescents (11–14 years old) from a school. IG: Five classrooms (*n* = 93); CG: six classrooms (*n* = 97). **PA and SB measurements**: PA: Accelerometry. SB: No measured.	**Supporting theory**: SDT and self-persuasion theory. **Social function promoted**: Social influence and social modeling. **Intervention**: 1-week intervention. Influence agents (team captains) were nominated by peers and trained via smartphones to implement the intervention, with daily follow-up. The influence agents were trained to promote PA in the classroom through four strategies: Social facilitation, modeling, example and acting, and impression management. The control group received no intervention. **SNI approach**: Individual. **Effects**: No effects on PA
Woudenberg, 2020 [27] Netherlands	**Study design**: Clustered RCT. **Sample characteristics**: 446 children and adolescents (9–16 years old) from 11 schools. (IG “social network”: 7 classes *n* = 131; IG “mass media”: 7 class *n* = 123; CG: 12 class *n* = 192). **PA and SB measurements**: PA: Accelerometry. SB: No measured.	**Supporting theory**: Theory of planned behavior and SDT. **Social function promoted**: Social influence and social norms. **Intervention**: 1-week intervention. Influence agents were nominated by peers and trained to implement the intervention, creating PA vlogs based on peers’ different social influence components. They did not receive training in how they can influence the PA of peers. In the mass media intervention, participants were exposed to vlogs made by unfamiliar peers. The control group received no intervention. **SNI approach**: Individual. **Effects**: No effects on PA
Jago, 2021 [33] England	**Study design**: Cluster RCT. **Sample characteristics**: 1558 girls (13–14 years old) from 20 state-funded secondary schools from three broad regions. IG: 10 schools (*n* = 758); CG: 10 schools (*n* = 800). **PA and SB measurements**: Accelerometry	**Supporting theory**: DOI and SDT. **Social function promoted**: Social support. **Intervention**: 10-week intervention based on the ASSIST intervention model. Peer supporters were identified by nominations and then trained for three days to play a supporter role within their classrooms to promote PA among their peer group. The training covered PA importance, how to be active, initiate conversations with peers about PA, and encourage peers to be active. The CG received no intervention. **SNI approach**: Individual. **Effects**: No effects on PA or SB
Sebire, 2018 [34] England	**Study design**: Feasibility RCT. **Sample characteristics**: 427 girls (12–13 years old) from six state more deprived secondary schools. Fifty-three were trained as peer supporters. IG = 4 schools (*n* = 269); CG: 2 schools (*n* = 158) **PA and SB measurements**: Accelerometry	**Supporting theory**: DOI and SDT. **Social function promoted**: Social influence. **Intervention**: 10-week intervention based on the ASSIST intervention model. Briefly, it comprised (a) peer-supporter nomination, (b) a train-the-trainers program, and (c) peer-supporter training followed by a 10-week informal PA message diffusion period. Control schools did not receive the intervention. **SNI approach**: Individual. **Effects**: Intervention had the potential to positively affect adolescent girls’ MVPA and reduce ST compared to controls 4–5 months post-intervention.
Barr-Anderson, 2012 [35] USA	**Study design**: Quasi-experimental feasibility study. **Sample characteristics**: 148 6th-grade students (age mean 11.2) from 4 schools in metropolitan areas with racially/ethnically diverse students. IG: 2 schools (*n* = 87); CG: 2 schools (*n* = 61). **PA and SB measurements**: PA: Previous Day Physical Activity Recall (PDPAR). SB: not measured.	**Supporting theory**: SCT. **Social function promoted**: Social influence, social support, and social norms. **Intervention**: 6-week intervention. The two intervention schools received the enhanced PALA + Peers program. The intervention included DVD of PA made by peers to encourage PA, training peer-led (*n* = 28) nominated to lead classroom sessions with teachers, PA, and healthy eating. Control schools received the standard PALA program. **SNI approach**: Individual. **Effects**: The intervention was successful in increasing moderate PA in all students and MVPA in girls

*RCT* randomized controlled trial, *PA* physical activity, *MVPA* moderated-vigorous physical activity, *IG* intervention group, *CG* control group, *SB* sedentary behavior, *ST* sitting time, *SNI* social network intervention, *SCT* social cognitive theory, *DOI* diffusion of innovation theory, *SDT* self-determination theory, *ASSIST* A Stop Smoking in Schools Trial, *PALA* Presidential Active Lifestyle Award

### Interventions details

The length of the interventions varied from 1-week [27, 28] to 10 weeks [32–34]. Intervention strategies consisted of forming same-sex groups to obtain rewards related to PA levels of friends by direct and indirect reciprocity, collective and individual levels of PA, and random reward [29], training peer-nominated peers to promote, disseminate information for PA in the classroom [28], serving as support for increase PA and decrease SB [32–34], and creating PA-related digital content as vlogs [27] and CDs [35] for classmates. Two studies were conducted only on girls [33, 34]. The intervention model ASSIST (A Stop Smoking in Schools Trial) [36] was adapted to address PA and SB in three studies [32–34]. Six out of seven studies were based on individual approaches of SNI [27, 28, 32–35]. Two studies were effective in increasing PA [29, 35], and one in increasing PA and reducing SB [34]. Characteristics of the SNI are shown in Table 1.

### Theoretical support for interventions and social function promoted

All studies reported a theoretical framework. These theories included Self-determination theory (SDT) with Self-persuasion theory (SPT) [28], SDT with planned behavior theory (PBT) [27], diffusion of innovation theory (DOI theory) [32], SDT and DOI theory [33, 34], social cognitive theory (SCT) [35], and social network theory [29]. The most frequent social functions or mechanisms promoted were social influence [27–29, 32, 34, 35], followed by social support [32, 33, 35], social norms [27, 29, 37], social modeling [28], and social pressure [29].

### Dimensions of external validity

#### Replicability

The intervention’s replicability was analyzed by TIDieR, and the summary is depicted in Table 2. All studies reported the items “Name”, “Why”, “What procedures”, “What materials”, “Who provided”, “How” and “When and how much”. The less reported items were “Tailoring”, “Modification”, and “How well actual”. The items “Where” and “How well planned” were reported in four and five studies, respectively. The most complete study reported 11/12 items [32], followed by 10/12 items [35]. Two studies reported 9/12 items [27, 28]. The other three reported 8/12 items [29, 33, 34].

**Table 2:** Summary of TIDieR items described in the analyzed studies

Study	TIDieR Criteria												Total score
	Name	Why	What		Who provided	How	Where	When and how much	Tailoring	Modifications	How well		
			Materials	Procedures							Planned	Actual	
Proestakis, 2018 [29]	1	1	1	1	1	1	0	1	0	0	1	0	8/12
Bell, 2017 [32]	1	1	1	1	1	1	1	1	1	0	1	1	11/12
Woudenberg, 2018 [28]	1	1	1	1	1	1	1	1	0	0	1	0	9/12
Woudenberg, 2020 [27]	1	1	1	1	1	1	1	1	0	0	1	0	9/12
Jago, 2021 [33]	1	1	1	1	1	1	1	1	0	0	0	0	8/12
Sebire, 2018 [34]	1	1	1	1	1	1	0	1	0	1	0	0	8/12
Barr-Anderson, 2012 [35]	1	1	1	1	1	1	0	1	0	1	1	1	10/12
**Total studies**	7	7	7	7	7	7	4	7	1	2	5	2	

#### Applicability

Precis-2 is a tool that evaluates how pragmatic or explanatory a study is as an indicator of SNI applicability. The nine items were assessed in each study, and the results are shown in Table 3. Very pragmatic items reported were Eligibility, Recruitment, and Primary outcome, all with a mean = 5.0. Rather pragmatic items reported were Flexibility of adherence (mean = 4.7) and Setting (mean = 4.6). Items considered equally pragmatic and explanatory were Flexibility of delivery (mean = 3.7) and Follow-up (mean = 3.1). The items reported as rather explanatory were Organisation (mean = 2.3) and Primary analysis (mean = 2.1). There were no items reported as very explanatory.

**Fig. 2:**
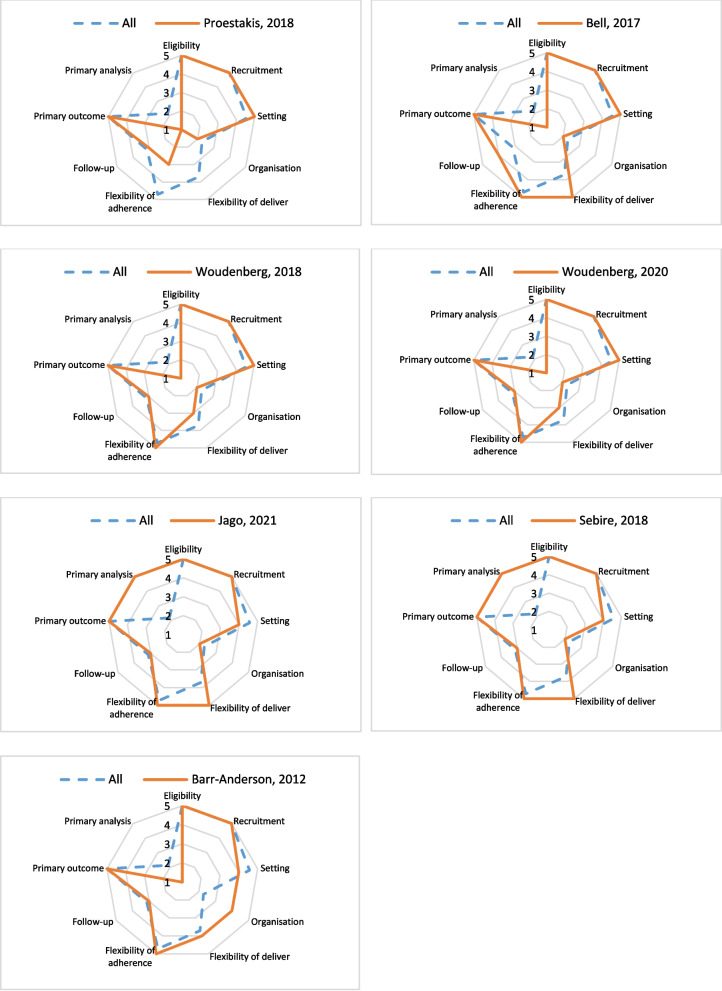
PRECIS-2 wheels for the analyzed studies

Six studies reached a Precis-2 score close to 4 or higher (between 3.8 and 4.3), indicating that these studies were Rather pragmatic [27, 28, 32–35]. The other study, with a Precis-2 score of 3.3, was classified as equally pragmatic and explanatory [29]. No studies were considered very explanatory, rather explanatory, or very pragmatic. That indicates that social network intervention tends to be pragmatic. The score of each study is compared with the general average of all the studies in each item of Precis-2 (Fig. 2).

**Table 3:** Summary of PRECIS-2 scores for the analyzed studies

Study	Eligibility	Recruitment	Setting	Organisation	Flexibility of deliver	Flexibility of adherence	Follow-up	Primary outcome	Primary analysis	Precis 2 ave score
Proestakis, 2018 [29]	5	5	5	2	1	3	3	5	1	3.3
Bell, 2017 [32]	5	5	5	2	5	5	4	5	1	4.1
Woudenberg, 2018 [28]	5	5	5	2	3	5	3	5	1	3.8
Woudenberg, 2020 [27]	5	5	5	2	3	5	3	5	1	3.8
Jago, 2021 [33]	5	5	4	2	5	5	3	5	5	4.3
Sebire, 2018 [34]	5	5	4	2	5	5	3	5	5	4.3
Barr-Anderson, 2012 [35]	5	5	4	4	4	5	3	5	1	4.0
**All (mean)**	5.0	5.0	4.6	2.3	3.7	4.7	3.1	5.0	2.1	

#### Generalizability

**Fig. 3:**
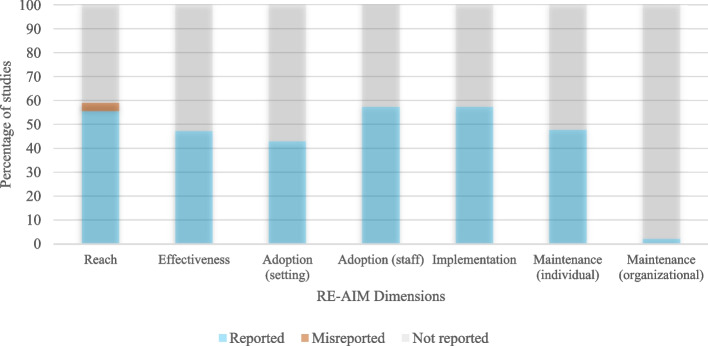
Summary of reporting RE-AIM dimensions

Generalizability was assessed with the RE-AIM tool. In each RE-AIM dimension, a percentage was made according to the number of items reported in each dimension. The analysis by dimensions indicated that the less reported dimension was maintenance (organizational); only one study reported items related to this dimension [29]. Low percentages, under 50%, were reported in the dimensions of effectiveness, adoption (setting), and maintenance (individual). The dimensions of Reach, Adoption (staff), and Implementation had percentages above 50% (see Fig. 3).

In each study, the percentage of compliance with each dimension and an average of all the dimensions together were analyzed (see Table 4). The maximum RE-AIM score was 61.2% [34], followed by 55.4% [27], and 51.7% [33]. Only these three studies had a RE-AIM score above 50%. The minimum RE-AIM scores were 26.2% [35] and 29.5% [29]. The other two studies had RE-AIM scores of 38.7% [28] and 46.6% [32].

**Table 4:** Percentage of completeness report of RE-AIM dimensions in the analyzed studies

Study	Reach	Effectiveness	Adoption (setting)	Adoption (staff)	Implementation	Maintenance (individual)	Maintenance (organizational)	Mean
Proestakis, 2018 [29]	25	30	37.5	22.2	44.4	33.3	14.3	29.5
Bell, 2017 [32]	37.5	40	37.5	66.7	77.8	66.7	0.0	46.6
Woudenberg, 2018 [28]	62.5	50	25	66.7	33.3	33.3	0.0	38.7
Woudenberg, 2020 [27]	75	50	62.5	77.8	55.6	66.7	0.0	55.4
Jago, 2021 [33]	75	70	50	66.7	33.3	66.7	0.0	51.7
Sebire, 2018 [34]	75	80	62.5	66.7	77.8	66.7	0.0	61.2
Barr-Anderson, 2012 [35]	37.5	10	25	33.3	77.8	0.0	0.0	26.2

#### Integration/intervention details

Following the recommendations for specifying and reporting implementation strategies in intervention studies, the evaluated dimensions of external validity were integrated with the domains specified to operationalize strategies: The actor, the action, action targets, temporality, dose, implementation targets affected, and theoretical justification [20]. Table 5 depicts each domain according to reproducibility, applicability, and generalizability purposes.

**Table 5:** Domains of implementation strategies for dimensions of external validity of social network intervention studies

Domains of implementation strategies	Dimensions of external validity		
	Replicability	Applicability	Generalizability
Actor	Members of school social networks were those who enacted the strategies.	Pragmatic since the actors of interventions were students in usual education settings.	The analyzed SNI studies were based on already established school social networks.
The action	Students received information to generate peer pressure or training to be supporters and leaders, to disseminate information, and to influence PA and SB positively.	Explanatory, considering that some of the SNI were implemented with the assistance of non-school staff, something that only sometimes occurs in schools. However, most of the activities may be implemented by school staff and are aligned with the usual strategies used in school settings.	The domains of adoption (staff) and implementation of SNI were moderately well described. However, adoption and implementation costs were not frequently reported.
Action targets	Actors may require training to perform their assigned roles within the social network. The training was focused on increasing social skills and knowledge.	Pragmatic because the studied SNI takes advantage of learning social skills fostered in the classroom and the social interactions that naturally occur within schools.	The theories used to develop the analyzed SNI offer a wide range of social mechanisms to promote the intended behavior: social influence, social support, social modeling, social pressure, and social norms.
Temporality	The duration of the SNI ranged from one week to 10 months, and training stages were reported.	The analyzed SNI were more pragmatic than explanatory because temporality is easily covered during a usual school year.	Although in most of the studies, the temporality of the SNI was reported, the frequency and content of the social interactions that take place within the school social networks are challenging to control and monitor.
Dose	The number of sessions or contacts ranged from two contacts per week to one per month. In some studies, this information needed to be provided.	Pragmatic because the highest dose of the studied SNI is easily reached in usual school settings.	The reported dose applies to the traditional schedule of school settings.
Implementation outcomes	Strategies for intervention fidelity and assessment of fidelity should be improved in the report of SNI.	Most of the analyzed SNI reported high flexibility of adherence and implemented non-mandatory activities; therefore, analyzed SNI were pragmatic.	Most SNI did not report enough information about three implementation outcomes: adoption, costs, and maintenance.
Theoretical justification	All the studied SNI reported theoretical justification.	This domain is not explored in the PRECIS-2 tool.	All the studied SNI reported theoretical justification. In addition, all the studies explored characteristics of the already and naturally established social networks within the schools. This examination is a critical key in SNI and is considered a “determinant of practice”.

## Discussion

This scoping review aimed to evaluate and synthesize the implementation process of social network interventions to modify physical activity and sedentary behaviors in childhood and adolescence. Several findings were identified in the scoping review and are presented in the following sections. The analyzed SNI for PA and SB have been reported with high replicability (TIDieR), were conducted pragmatically, i.e., with very similar conditions to real settings (PRECIS-2), and the report of some RE-AIM domains support the generalizability of SNI.

### Theoretical support for interventions

The most frequent theoretical framework reported in the interventions was the self-determination theory (SDT), used in four out of the seven analyzed studies along with other theories [27, 28, 33, 34]. The SDT is based on intrinsic or autonomous motivation and psychological needs as relation, autonomy, and competence [38]. Recently, a systematic review found that SDT-based interventions improved different health domains [39]. In children and adolescents, the evidence suggests that SDT constructs help to explain physical activity behavior [40].

Diffusion of innovation theory (DOI) was used as a theoretical framework in other social network interventions [32–34]. DOI theory is considered relevant for social network interventions [16] since it is helpful to explain how new ideas and practices spread within the social network [41, 42]. Likewise, integrating the theory of social networks with DOI theory has been suggested to improve the implementation of interventions, achieving a comprehensive approach [43]. In the present scoping review, two interventions applied a combination of SDT and DOI theory [33, 34]. However, only the study of Sebire et al. (2018) positively affected PA and SB.

Other theories, such as social cognitive theory (SCT), theory of planned behavior (TPB), and self-persuasion theory (SPT), were used to inform the interventions. However, only one study reported positive effects on PA [35]. However, SCT has been previously used in interventions for PA and obesity with no significant results, and low efficacy [44, 45]. The theoretical support for SNI is required not only for identifying those constructs to intervene during the implementation process and how they interact with each other to influence the outcome of interest but also, more importantly, for helping to explain how patterns of the social system may be modified by fostering or intensifying some social interaction mechanisms to optimize the system behavior. This perspective implies the adoption of the systems lens for using theoretical frameworks for complex interventions [46].

### Interpersonal relationships promoted

The social mechanism most promoted in the studies included in this review was social influence, analyzed in all the studies except for the study of Jago et al. [33]. This finding is consistent with what has been pointed out in the literature about the role of social influence in the relationship between health behavior and social networks [47]. The evidence shows that social influence is positively associated with health behaviors in school adolescents [9] and that individual PA levels may result from the influence of friends and peer [12, 14, 15]. The other social mechanisms reported in the studies included social norms, social pressure, social support, and social modeling. The identification of these mechanisms is a key step to define the implementation of strategies [8]. However, it is difficult to determine the actual social mechanisms responsible for behavioral changes due to social networks’ inherent, complex dynamics and their natural evolution [47].

### Types of social network interventions

The identification of the social network intervention approach used is an important aspect to understand the implementation process, since each typology of SNI describes different strategies to be implemented [16]. Six out of seven studies were based on individual approaches of SNI. Generally, researchers used friend’s nomination to identify the agents by a network parameter (centrality) and then trained them to implement strategies within the network for behavior change; two of these studies had positive effects [34, 35]. This type of SNI is supported by the most substantial evidence of effectiveness in the adult population [8]. The study that used a social approach based on the specific type of induction and alteration based on reciprocity positively affected the PA level [29]. A review of SNI for health behaviors in adults identified that the social approach, especially induction, was the most frequent SNI typology used [8].

In the present review, two interventions used “closeness centrality”: influence agents closely connected to all other network members [27, 28]. Four interventions used “indegree centrality”: influence agents who received the most nominations or were popular in the network [32–35]. This strategy is supported by the fact that popularity in social networks has been associated with engagement in health behaviors and significant associations for predicting health behavior [9]. However, the SNI type’s effectiveness for behavior change still requires more research attention.

It is important to note that previous evidence syntheses have also explored PA interventions based on peer-to-peer approaches [48, 49]. Studies analyzed in these reviews applied diverse peer-leadership approaches, where leaders were trained to foster PA among peers. In these studies, the peer leader was identified using different criteria than those applied in SNI, i.e., applying teacher criteria or based on the student’s leadership skills. The difference with these reviews is that our scoping review focuses on those interventions in which sociometric data from networks is used to learn from the community, a particular characteristic of SNI [16]. In other words, the difference lies in the methods used to identify who delivers the strategies. For this review, we strictly adhere to the definition of SNI, in which network information is required to inform the design of the intervention [16].

It is worth noting that four of the seven studies were identified using manual search and previous knowledge of the researchers. This may be explained by the fact that these studies do not include the term of social network intervention in the title or abstract. However, in the methods section, authors describe the procedures that fit with the operational definition of SNI [32–35].

### External validity: replicability

In this scoping review, the replicability of interventions was analyzed by the TIDieR tool, and most of the items were appropriately reported. In general, all the SNI had good replicability scores. However, “Tailoring”, “Modification”, and “How well actual” were the items with the lowest frequency of reporting, crucial aspects for assessing intervention fidelity, adherence, and adverse events. These items are useful to complete the description of the implementation process and should be addressed in future studies. Similar findings about frequencies of complete reports have been documented in overviews of systematic reviews [50, 51]. Even though the analyzed studies had acceptable replicability, more complete and accurate reporting could reduce research waste, improve evidence synthesis, and implementation in other contexts [52].

### External validity: applicability

Applicability was assessed with Precis-2; in general, SNI tended to be pragmatic. There were no studies considered rather explanatory or very explanatory. Social network interventions have the particularity of using established networks, in this case, the classrooms. This allows many elements to be pragmatic. In this review, the most pragmatic items were “Eligibility”, “Recruitment”, and “Primary outcome”. At the same time, “Follow-up” tends to be equally pragmatic and explanatory, and “Organisation” and “Primary analysis” tend to be rather explanatory. These results differ from other reviews where “Primary analysis” tends to be more pragmatic [53, 54], and “Eligibility”, “Organisation”, and “Follow-up” tend to be more explanatory in primary health care [53, 54].

In this review, two studies considered pragmatic positively affected PA [34, 35]. However, the most explanatory study also positively improved PA in adolescents in SNI [29]. A systematic review concluded that pragmatic trials were ineffective in improving PA in children [55], and others concluded that more pragmatic studies were associated with smaller increases in PA [56]. It is worth mentioning that in most cases, SNI has aspects that are inevitably considered explanatory in social networks. For instance, although the interventions were implemented in usual school settings, characteristics of the social network of students are not commonly analyzed to inform pedagogical interventions. Therefore, this exploration could be considered explanatory.

### External validity: generalizability

Generalizability was assessed with RE-AIM. Overall, three RE-AIM domains (reach, adoption (staff), and implementation) support an acceptable level of the generalizability of findings. The report of the rest of the dimensions needs to be improved, particularly maintenance (organizational). The percentage mean for studies varied from 26.6% [35] to 61.2% [34]. The most reported dimension was reach with 55%, adoption (staff) and implementation both with 57%, and the least reported was maintenance (organizational) with only 2%. Previous systematic reviews have also found that Reach is the dimension where more items are reported in PA school interventions among adolescents [57]. In specific populations, such as indigenous youth, the result was different. A review concluded that the dimensions of reach and implementation were the most poorly reported in PA interventions [58]. However, items could have been better described with more information [57], while the dimension of maintenance is the most poorly reported [59]. In this regard, it has been pointed out that maintenance is one of the most challenging elements for physical activity interventions in schools since it requires the integration of multi-stakeholder perspectives from classroom, school, and policy levels [60].

### External validity: integration

All the analyzed studies were conducted in schools, reinforcing the premise that the school setting offers a good opportunity to promote health and prevent disease [61]. In the analyzed SNI studies, students enacted the strategies deployed in their social networks in real-world education settings. Social interactions are the core of SNI. However, students should sometimes receive training to improve their social skills and unfold social mechanisms to promote the intended behaviors. Since social learning skills are part of the learning objective in school settings, SNI fits the requirements for implementation within schools. Although the analyzed studies specify the intervention time, the exposure dose, and other relevant aspects for external validity, social interactions occur naturally during school hours and are not restricted to educational intentions. Therefore, it is a challenge to effectively register the quantity and quality of social interactions that may occur during school days that have yet to be planned within the study. In addition, there is evidence of the effectiveness of SNI in the short and long term (more or less than six months) in some health-related behaviors [8]. All the studies analyzed in the present scoping review had a duration of no more than ten weeks of intervention, and three of them reported positive effects on PA in interventions between 6 and 10 weeks [29, 34, 35]. The SNI for PA and SB tend to be conducted with high external validity in the domains of actor, action targets, temporality, dose, and theoretical justification. The domains of action and implementation outcomes need to be improved in the reports for SNI replicability, applicability, and generalizability.

It is worth indicating that when integrating the three dimensions of external validity according to the domains proposed to describe the implementation of strategies [20], there is a risk of omitting relevant aspects of each intervention. This, added to the need to attend to contextual conditions, as suggested from the systems perspective [18], implies that a reading of the context must be carried out to adapt and contextualize the implementation of the strategies.

The findings of this scoping review contribute to the initiative of whole systems approaches for physical activity promotion [62] because tools such as social network analysis are used to understand systems as a central issue within implementation science [17]. Its application by public health and educational practitioners requires adopting a system approach [46], e.i., embracing the uncertainty and unpredictable nature of the relationship between actions and their consequences. From this perspective, SNI needs to be understood as events within the system [63]. Since schools are complex social systems [64], SNI requires identifying particular social dynamics within the network and being attentive enough to recognize changes produced by the system during the intervention [46]. For instance, the identification of central actors within the social network may be used to activate “leverage points” [65] that generates meaningful changes in the social systems of students [66]. In this regard, integrating context- and practice-based evidence is encouraged. Regarding implications for research, the present review’s findings help identify aspects to be explored in future research. Among these aspects, examining social network mechanisms and studies about the effectiveness and implementation processes of the SNI typologies, for both PA and SB, are elements to study in greater depth. Also, future research should be focused on SNI as a complementary component along with other participatory strategies and system mapping methods to better align with the whole system approach.

This review presents some limitations. First, the researchers used tools to evaluate replicability, applicability, and generalizability. However, this could have been more accurate if the program implementers had participated in the task. Second, all the SNI analyzed were conducted in school settings. The present review did not identify other spheres of socialization, such as neighborhoods, sports teams, churches, and the like. A third limitation is that in this scoping review, and as part of the narrative description of findings about the implementation process, the direction of the effect of the interventions has been mentioned, but not extensively analyzed. This procedure is usually done in systematic reviews of the effectiveness of interventions, as previously conducted in the field of SNI [8]. Among the strengths, integrating the three tools used as dimensions of external validity gave a novel approach to evaluate intervention studies from the D&I research perspective. Also, considering that diverse local circumstances define social network processes, structures, and dynamics, it is challenging to generalize these interventions. In this regard, the present scoping review highlights essential aspects of SNI. Endeavors to scale up SNI must pay special attention in the implementation process to identify these local circumstances responsible for the social dynamics unfolding in the networks.

## Conclusions

All the analyzed SNI were theory-driven interventions, and most were based on the combination of SDT with other theories like diffusion of innovation theory, self-persuasion theory, and theory of planned behavior. Other supporting theories were social cognitive theory and social network theory. The most frequent content of the interpersonal relationships or social mechanisms fostered in the studies was social influence, and most of the studies were based on individual approaches of SNI. The analyzed SNI for PA and SB in adolescents tend to be reported with high replicability. These studies were conducted pragmatically, i.e., with very similar conditions to real settings. And the RE-AIM domains of reach, adoption (staff), and implementation support the generalizability of SNI, while the report of the domain of maintenance (organizational) needs to be improved. Some domains of the principles of implementation strategies of SNI had acceptable external validity (actor, action targets, temporality, dose, and theoretical justification). The domains of action and implementation outcomes need to be improved in the reports for SNI replicability, applicability, and generalizability.

### Supplementary Information

**Supplementary Material 1.**

**Supplementary Material 2.**

## Data Availability

The datasets used and/or analyzed during the current study are available from the corresponding author on reasonable request.

## Abbreviations

D&I: Dissemination and implementation research
PA: Physical activity
SB: Sedentary behavior
SNI: Social network intervention
JBI: Jhoana Briggs Institute
PCC: Population, concept, and context
TIDieR: Template for Intervention Description and Replication framework
PRECIS-2: Pragmatic-Explanatory Continuum Indicator Summary framework
RE-AIM: Reach, effectiveness, adoption, implementation, and maintenance framework
PRISMA-ScR: Preferred Reporting Items for Systematic reviews and Meta-Analyses extension for Scoping Reviews
SDT: Self-determination theory
SPT: Self-persuasion theory
PBT: Planned behavior theory
DOI: Diffusion of innovation theory
SCT: Social cognitive theory
