# Supplementary material for: Implementation processes of social network interventions for physical activity and sedentary behavior among children and adolescents: a scoping review
Source: BMC Public Health. 2024 Apr 22;24:1101. doi: 10.1186/s12889-024-18615-6 (PMC11034017; doi:10.1186/s12889-024-18615-6)
Supplement: Supplementary file 1 — Supplementary Material 1. [file 12889_2024_18615_MOESM1_ESM.docx]

Supplementary table S1. Search strategy

| **Variable** | **Mesh term** | **Related terms** |
| --- | --- | --- |
| Physical activity | Exercise | Physical exercise, sport |
| Sedentary behavior | Sedentary behavior | Sedentary behaviour, sitting, screen time, screen use, sedentary time, sedentary lifestyle. |
| Social network intervention | Social network analysis | "network intervention" OR “network-based intervention” OR “social network intervention” OR “friends intervention” |
| **Database** | **Syntax** | |
| ERIC | (("Social network analysis") OR ("network intervention") OR ("network-based intervention") OR ("social network intervention") OR ("friends intervention")) AND ("Physical activity") OR (sport) OR ("Sedentary behavior") OR ("Sedentary behaviour") OR (sitting) OR ("screen time") OR ("screen use") OR ("sedentary time") OR ("sedentary lifestyle") | |
| EBSCO | TI ( "Sedentary behavior" OR "Sedentary behaviour" OR "Sedentary lifestyle" OR sitting OR "screen time" OR "screen use" OR "Sedentary time" OR "Physical activity" OR sport ) OR AB ( "Sedentary behavior" OR "Sedentary behaviour" OR "Sedentary lifestyle" OR sitting OR "screen time" OR "screen use" OR "Sedentary time" OR "Physical activity" OR sport ) AND TI ( "social network analysis" OR "network intervention" OR "network-based intervention" OR "social network intervention" OR "friends intervention" ) OR AB ( "social network analysis" OR "network intervention" OR "network-based intervention" OR "social network intervention" OR "friends intervention" ) | |
| EMBASE | ('social network analysis' OR 'network intervention' OR 'network based intervention' OR 'social network intervention' OR 'friends intervention') AND ('physical activity' OR sport OR 'sedentary behavior' OR 'sedentary behaviour' OR sitting OR 'screen time' OR 'screen use' OR 'sedentary time' OR 'sedentary lifestyle') | |
| SCOPUS | TITLE-ABS-KEY ( "Social network analysis" OR "network intervention" OR "network-based intervention" OR "social network intervention" OR "friends intervention" ) AND TITLE-ABS-KEY ( "Physical activity" OR sport OR "Sedentary behavior" OR "Sedentary behaviour" OR sitting OR "screen time" OR "screen use" OR "sedentary time" OR "sedentary lifestyle") | |
| Lilacs | (("Social network analysis") OR ( "network intervention") OR ("network-based intervention") OR ("social network intervention") OR ("friends intervention")) AND (("Physical activity") OR (sport) OR ("Sedentary behavior") OR ("Sedentary behaviour") OR ( sitting) OR ("screen time") OR ("screen use") OR ("sedentary time") OR ("sedentary lifestyle")) | |
