# Supplementary material for: Implementation processes of social network interventions for physical activity and sedentary behavior among children and adolescents: a scoping review
Source: BMC Public Health. 2024 Apr 22;24:1101. doi: 10.1186/s12889-024-18615-6 (PMC11034017; doi:10.1186/s12889-024-18615-6)
Supplement: Supplementary file 2 — Supplementary Material 2. [file 12889_2024_18615_MOESM2_ESM.docx]

Supplementary table S2. Items and descriptions evaluated in the TIDieR, PRECIS-2, and RE-AIM tools.

| *Items* | *Description* |
| --- | --- |
| TIDieR |  |
| *Name* | Name or a phrase that describes the intervention. |
| *Why* | Any rationale, theory, or goal of the elements essential to the intervention. |
| *What materials* | Any physical or informational materials used in the intervention, including access to materials. |
| *What procedures* | Activities implemented in the intervention. |
| *Who provided* | Characteristics of each category of intervention provider. |
| *How* | Modalities of delivery. |
| *Where* | Location(s) where the intervention was implemented. |
| *When and how much* | Duration of intervention (number of sessions, schedule, duration, intensity, or dose). |
| *Tailoring* | If the intervention was planned to be personalized, titrated, or adapted (what, why, when, and how). |
| *Modification* | If changes were made during the intervention (what, why, when, and how). |
| *How well planned* | Assessment of adherence or fidelity (how and by whom, and strategies used to maintain or improve fidelity). |
| PRECIS-2 |  |
| *Eligibility criteria* | Similarities between the trial participants and those who would receive this intervention. |
| *Recruitment* | Efforts to recruit participants compared to what is done in usual care to engage patients. |
| *Setting* | Differences between the settings of the trial and the usual care setting. |
| *Organisation* | Differences between the resources, provider expertise, and the organisation of care delivery in the intervention and those available in usual care. |
| *Flexibility delivery* | Differences in the flexibility of how the intervention is delivered and the flexibility anticipated in usual care. |
| *Flexibility adherence* | Differences in the flexibility of how participants are monitored and encouraged to adhere to the intervention and the flexibility anticipated in usual care. |
| *Follow-up* | Differences in the intensity of measurement and follow-up in the trial and the typical follow-up in usual care. |
| *Primary outcomes* | Relevance of the trial’s primary outcome to participants. |
| *Primary analysis* | Inclusion of all data in the analysis of the primary outcome. |
| RE-AIM |  |
| *Reach (8 items)* | Target population, demographic and behavioral information of target population, method to identify the target population, recruitment strategies, inclusion and exclusion criteria, sample size, participation rate, and characteristics of both participants and non-participants. |
| *Effectiveness (10 items)* | Effect of the intervention in the primary outcome, report of mediators, report of moderators, intent-to-treat or present at follow-up, imputation procedures, quality of life measure, measure unintended consequences (negative) and results, percent attrition (at program completion), cost-effectiveness, use of qualitative methods to measure efficacy/effectiveness. |
| *Adoption setting (8 items)* | # Of eligible and invited (exposed) settings, # participating, participation rate, description of the targeted location, inclusion/exclusion criteria of setting, description of intervention location, method to identify setting, average # of persons served per setting. |
| *Adoption staff (9 items):* | # Eligible and invited staff, # of staff participating in delivery, staff participation rate, method to identify target delivery agent, level of expertise of delivery agent, inclusion/exclusion criteria of delivery agent, measures of cost adoption, dissemination beyond planned initially, use of qualitative methods to measure adoption. |
| *Implementation (9 items)* | Theories, intervention number of contacts, timing of contacts, duration of contacts, extent protocol delivered as intended (%), consistency of implementation across settings, participant attendance/completion rates, measure of cost, use of qualitative methods to measure implementation. |
| *Maintenance Individual (3 items)* | Was individual behavior assessed at some duration following completion of the intervention? Attrition, use of qualitative methods to measure individual methods. |
| *Maintenance Organization (7 items)* | Report alignment with organization mission, is the program still in place? If no, reason for discontinuation? If yes, was the program modified? Was the program institutionalized? Attrition, use of qualitative methods to measure organizational maintenance |
